# Sex and chronic stress alter delta opioid receptor distribution within rat hippocampal CA1 pyramidal cells following behavioral challenges

**DOI:** 10.1016/j.ynstr.2020.100236

**Published:** 2020-06-22

**Authors:** Batsheva R. Rubin, Megan A. Johnson, Jared M. Berman, Ellen Goldstein, Vera Pertsovskaya, Yan Zhou, Natalina H. Contoreggi, Andreina G. Dyer, Jason D. Gray, Elizabeth M. Waters, Bruce S. McEwen, Mary Jeanne Kreek, Teresa A. Milner

**Affiliations:** a Feil Family Brain and Mind Research Institute, Weill Cornell Medicine, 407 East 61st Street, New York, NY, 10065, United States; b The Laboratory of the Biology of Addictive Diseases, The Rockefeller University, 1230 York Avenue, New York, NY, 10065, United States; c Harold and Margaret Milliken Hatch Laboratory of Neuroendocrinology, The Rockefeller University, 1230 York Avenue, New York, NY, 10065, United States

**Keywords:** Delta opioid receptor, Electron microscopy, Pyramidal cells, Oxycodone associative-learning

## Abstract

Following oxycodone (Oxy) conditioned place preference (CPP), delta opioid receptors (DORs) differentially redistribute in hippocampal CA3 pyramidal cells in female and male rats in a manner that would promote plasticity and opioid-associative learning processes. However, following chronic immobilization stress (CIS), males do not acquire Oxy-CPP and the trafficking of DORs in CA3 pyramidal neurons is attenuated. Here, we examined the subcellular distribution of DORs in CA1 pyramidal cells using electron microscopy in these same cohorts.

**CPP:**

Saline (Sal)-females compared to Sal-males have more cytoplasmic and total DORs in dendrites and more DOR-labeled spines. Following Oxy-CPP, DORs redistribute from near-plasmalemma pools in dendrites to spines in males.

**CIS:**

Control females compared to control males have more near-plasmalemmal dendritic DORs. Following CIS, dendritic DORs are elevated in the cytoplasm in females and near-plasmalemma in males.

**CIS plus CPP:**

CIS Sal-females compared to CIS Sal-males have more DORs on the plasmalemma of dendrites and in spines. After Oxy, the distribution of DORs does not change in either females or males.

**Conclusion:**

Following Oxy-CPP, DORs within CA1 pyramidal cells remain positioned in naïve female rats to enhance sensitivity to DOR agonists and traffic to dendritic spines in naïve males where they can promote plasticity processes. Following CIS plus behavioral enrichment, DORs are redistributed within CA1 pyramidal cells in females in a manner that could enhance sensitivity to DOR agonists. Conversely, CIS plus behavioral enrichment does not alter DORs in CA1 pyramidal cells in males, which may contribute to their diminished capacity to acquire Oxy-CPP.

## Highlights

• Saline-injected (Sal) females had higher dendritic delta opioid receptor (DOR) levels.
• Oxycodone (Oxy) conditioned place preference (CPP) increased DORs in spines in males.
• Chronic immobilization stress (CIS) alone increased dendritic DORs in both sexes.
• DORs on the plasmalemma and in spines increased in CIS-Sal females exposed to CPP.
• Dendritic DOR distributions were unchanged in CIS Oxy-males that have attenuated CPP.

## Graphical abstract

**Figure:**
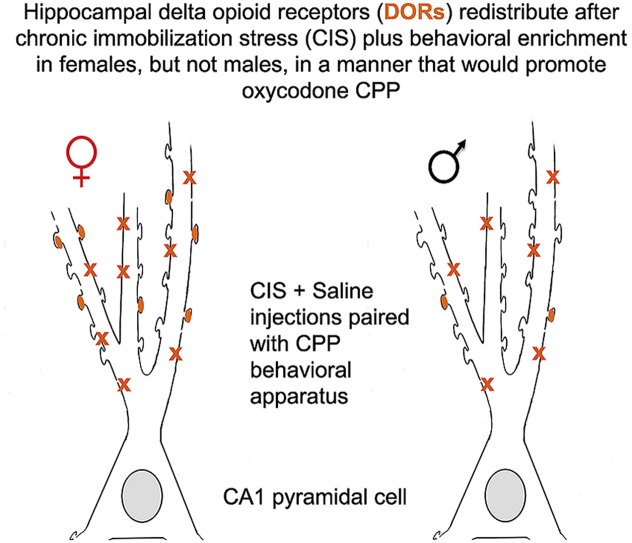

## Abbreviations

ABC: avidin-biotin complex
*Akt*: AKT serine/threonine kinase
*Arc*: activity regulated cytoskeletal-associated protein
BDNF: brain derived neurotrophic factor
BSA: bovine serum albumin
CIS: chronic immobilization stress
CPP: conditioned place preference
CRF: corticotropin releasing factor
CRFR1: corticotropin releasing factor receptor type 1
DAB: diaminobenzidine
DG: dentate gyrus
DOR: delta opioid receptor
GABA: Gamma-amino butyric acid
ir: immunoreactivity
LEnk: leu-enkephalin
LTP: long-term potentiation
Mapk1: mitogen activated protein kinase
MOR: mu opioid receptor
NPY: neuropeptide Y
Oxy: oxycodone
PARV: parvalbumin
PFA: paraformaldehyde
PB: phosphate buffer
PM: plasma membrane
ROI: region of interest
Sal: saline
SLM: stratum lacunosum-moleculare
SLu: stratum lucidum
SO: stratum oriens
SOM: somatostatin
SR: stratum radiatum
TAT: temporoammonic tract
TS: tris-buffered saline

## 1 Introduction

Stress is an important risk factor for addiction, a relationship of which extensive research over the past few decades has sought to elucidate (Sinha, 2008). Chronic stress impairs spatial learning and memory in male but not female rodents (Luine et al., 2007; McEwen, 1999; McEwen and Milner, 2017; Sousa et al., 2000) and learning and memory are important contributors in the addiction process (Becker et al., 2017). The associative learning process relies upon functional networks with the hippocampus, where chronic stress results in dendritic retraction in CA3 hippocampal pyramidal cells and the loss of parvalbumin-containing interneurons in male but not in female rodents [reviewed in (McEwen and Milner, 2017)]. In CA3 pyramidal cells, opioid signaling is important for contextual associative learning (Kesner and Warthen, 2010; Meilandt et al., 2004) and thus stress could differently alter processes important for opioid addiction acquisition processes in female and male rodents.

Our recent studies examining conditioned place preference (CPP) to the mu opioid receptor (MOR) agonist oxycodone (Oxy) have provided insight into the relationship between stress and associative learning processes important for opioid addiction. Although both female and male unstressed Sprague Dawley rats acquired Oxy CPP (Randesi et al., 2019; Ryan et al., 2018), examination of the opioid system in the hippocampus revealed that Oxy CPP redistributes opioid receptors in hippocampal circuits that would promote opioid-associative learning processes especially in females. For example, Oxy CPP in both females and males resulted in a redistribution of delta opioid receptors (DORs) to CA3 pyramidal cell dendritic spines (Ryan et al., 2018), positioning DORs to promote excitation as well as opioid-mediated long-term potentiation (LTP) (Harte-Hargrove et al., 2015). However, only in females were MORs and DORs redistributed to plasmalemmal compartments in GABAergic hilar interneurons in females (Ryan et al., 2018), positioning them to promote disinhibition of granule cells (Drake et al., 2007). As a consequence, disinhibition of MORs in parvalbumin (PARV) -containing basket interneurons could result in greater release of enkephalin from granule cells and indirectly contribute to opioid-mediated LTP at the mossy fiber-CA3 synapse (Harte-Hargrove et al., 2015). Moreover, disinhibition of DORs found in somatostatin (SOM)/neuropeptide Y (NPY) interneurons (Amaral et al., 1988; Commons and Milner, 1996; Milner and Bacon, 1989; Milner and Veznedaroglu, 1992) can inhibit NPY release and could promote LTP in leu-enkephalin (LEnk) -containing lateral perforant path afferents (Commons and Milner, 1995; Gall et al., 1981; Sperk et al., 2007).

In contrast to unstressed conditions, chronic immobilization stress (CIS) results in even greater sex differences in the hippocampal opioid system and Oxy CPP. CIS in males, but not females, down-regulates expression of opioid, stress and other signaling molecules in the hippocampus that are critical for modulating synaptic plasticity (Randesi et al., 2018) and alters the distribution and availability of opioid receptors in ways that favor reduction of addiction in males over females (Reich et al., 2019). Following CIS in females, LEnk levels in mossy fibers and DOR densities in their targeted CA3 pyramidal cell dendritic spines remain elevated (Mazid et al., 2016; Pierce et al., 2014). Moreover, similar to the redistribution of the MORs and DORs seen in unstressed females after Oxy CPP (Ryan et al., 2018), CIS in females increases plasmalemmal associated MORs and DORs on PARV interneuron dendrites (Mazid et al., 2016; Milner et al., 2013). In contrast to CIS females, CIS males have reduced levels of mossy fiber LEnk, decreased DORs in mossy fiber-CA3 pyramidal cell synapses, no change in the distribution of MORs in PARV-labeled interneurons and decreased plasmalemmal-associated DORs in GABAergic interneuron dendrites in the dentate gyrus (DG) (Mazid et al., 2016; Milner et al., 2013; Pierce et al., 2014). Thus, CIS sets up opioid circuits in the CA3 and DG in the females for enhanced sensitivity to opioid agonists whereas CIS essentially “shuts down” these circuits in males. These sex differences in the opioid system may contribute to the attenuation of Oxy CPP in male rats following CIS (Bellamy et al., 2019; Reich et al., 2019).

Our previous studies examining the effects of Oxy CPP in unstressed and CIS rats were concentrated in the CA3 and DG, while the effects on CA1 remain unknown. In CA1, DORs have been shown to affect excitatory transmission and the induction of synaptic plasticity in rat CA1 pyramidal cells both directly and indirectly through inhibition of interneurons (Bao et al., 2007; Rezai et al., 2013; Svoboda et al., 1999). Within the rat CA1, DOR-immunoreactivity and mRNA expression are present within pyramidal cells as well as scattered interneurons primarily in stratum oriens (Commons and Milner, 1997; George et al., 1994; Stumm et al., 2004; Williams et al., 2011b). Additionally, our prior studies showed that the subcellular distribution of DORs in CA1 is altered by sex and hormonal milieu similar to our findings in CA3 (Mazid et al., 2016; Williams et al., 2011b).

Compared to CA3, CA1 circuits have some similarities but also some differences. Both CA3 and CA1 are part of the trisynaptic hippocampal pathway which is important for hippocampal output [reviewed in (Scharfman and MacLusky, 2017)]. Moreover, both CA3 and CA1 receive enkephalin inputs from the entorhinal cortex (Drake et al., 2007; Gall et al., 1981) albeit from different pathways [reviewed in (Freund and Buzsáki, 1996)]. In CA1, inputs from the lateral entorhinal temporoammonic tract have been shown to recruit DORs to the CA1 in the mouse hippocampus (Rezai et al., 2013). CA3 pyramidal cells project back to the DG (Scharfman and MacLusky, 2017) and receive direct projections from granule cell mossy fibers (Drake et al., 2007). In contrast, CA1 pyramidal cells project to the subiculum and entorhinal cortex as well as other limbic cortical areas including the lateral septum and nucleus accumbens and are not contacted by mossy fibers [reviewed in (Freund and Buzsáki, 1996)].

In addition to connectivity differences, the CA3 and CA1 regions respond differently to ovarian hormone levels and stress depending upon sex. While elevated estrogen levels increase dendritic spines in CA1, the density of CA3 spines does not vary across the estrous cycle [reviewed in (Sheppard et al., 2019)]. Additionally, while estrogens increase synaptic proteins in both CA1 and CA3, the effects in CA3 are less robust (Brake et al., 2001). Following chronic stress, CA3 pyramidal cell dendrites in female rodents do not change while in male rodents, they undergo severe atrophy (McEwen, 1999; McEwen and Milner, 2017). In males, chronic stress also results in a loss of mossy fiber synapses [reviewed in (Krugers et al., 2010b)]. In males, some studies report dendritic atrophy in CA1 pyramidal cells (Lambert et al., 1998; Sousa et al., 2000) whereas others do not (Alfarez et al., 2008; Woolley et al., 1990). Prolonged stress exposure in males can reduce dendritic spines in both CA3 and CA1 pyramidal cells (Krugers et al., 2010a). Thus, although CA1 and CA3 pyramidal cells both receive LEnk inputs and contain DORs, the differences in synaptic connectivity as well as sex differences in response to the hormonal milieu and stress could affect their respective responses to opioids.

This study aimed to elucidate sex differences in the effects of stress and Oxy CPP on the distribution of DORs in CA1 using tissue immunolabeled from the same cohorts of rats used in our previous studies in CA3 and DG (Reich et al., 2019; Ryan et al., 2018). To separate out the effects of stress from Oxy CPP, tissue from a cohort of rats that underwent CIS alone (Mazid et al., 2016; McAlinn et al., 2018; Milner et al., 2013; Pierce et al., 2014) also was included.

## 2 Materials & methods

### 2.1 Animals

Male (275–325 gm) and female (225–250 gm) Sprague Dawley rats (RRID:RGD_734476; N = 72; 24 per cohort) from Charles River Laboratories (Wilmington, MA) were 2–3 months old upon arrival. Three separate cohorts, each containing 24 rats with 6 rats/group, were used for this study. Cohort 1. Saline-injected (Sal) naïve female and male rats or Oxy-injected naïve male and female rats that were subjected to CPP [used previously (Ryan et al., 2018)]. Cohort 2. Unstressed and CIS female and male rats [used in our previous studies (Mazid et al., 2016; McAlinn et al., 2018; Milner et al., 2013; Pierce et al., 2014)]. Cohort 3. CIS Sal-females and males or CIS Oxy-females and males that were subjected to CPP [used previously (Reich et al., 2019)]. In all three cohorts, rats were randomly assigned to the respective groups. Rats in cohorts 1 and 3 were single-housed, as they were used for CPP studies, while rats in cohort 2 were pair-housed, as they were only used for CIS studies, in R20 rat cages (10.5 in x 19 in x 8 in; Ancare, Bellmore NY) with a 12-h light/dark cycle (lights on 0600–1800) and *ad libitum* access to water and food. Rats were allowed to acclimate to the vivarium 1 week prior to initiating estrous cycling or experimental procedures. All animal procedures were approved by the Rockefeller University and Weill Cornell Medicine Institutional Animal Care and Use Committees and were in accordance with the 2011 8th edition of the National Institutes of Health guidelines for the Care and Use of Laboratory Animals.

### 2.2 Estrous cycle determination

Vaginal smear cytology (Turner and Bagnara, 1971) was used to determine estrous cycle stage. Rats in cohort 2 were estrous cycled daily between 9:00 and 10:00 a.m. for about 10–14 days. Only rats that demonstrated two consecutive 4–5 day estrous cycles were used. Females used for cohort 2 (CIS only) were in the estrus phase of the estrous cycle as confirmed with uterine weight and plasma serum estradiol levels via radioimmunoassay in our prior study (Milner et al., 2013). Estrous cycles of female rats in the CPP experiments (cohorts 1 and 3) were only determined on the day of euthanasia, after the rats were anesthetized and prior to aortic perfusion (described below). Female rats analyzed from cohorts 1 (Oxy CPP) and 3 (CIS Oxy CPP) were in the estrus phase of the estrous cycle as determined in our prior studies (Reich et al., 2019; Ryan et al., 2018).

### 2.3 Chronic immobilization stress

In cohort 2 (CIS only), control and CIS rats were housed in separate rooms. The rats in cohort 3 (CIS followed by Oxy CPP) were housed in the same room; in this case, the cages containing the control and CIS rats were kept in custom-built cabinets (Phenome Technologies Inc.) directly attached to the ventilation system and equipped with lamp timers that maintained a 12:12 light/dark cycle (lights on at 0600). Rats were subjected to CIS for 10 consecutive days (Lucas et al., 2007; Shansky et al., 2010). For this, rats were placed in plastic cone shaped polyethylene bags that contained a small breathing hole in the apex and a Kotex mini-pad inside the bag below the rats’ tails for urine collection. After the rats were placed in the bag and sealed in with tape around their tails, the rats were left for 30 min undisturbed.

### 2.4 Oxy CPP

Rats in cohorts 1 and 3 were subjected to Oxy CPP as previously described (Reich et al., 2019; Ryan et al., 2018). The same experimenters performed all behavioral testing at the same time each day (within 3 h of the onset of the light cycle). Behavioral experiments on cohorts of rats were staged over a period of 4 days so that euthanasia would occur between 9:00 a.m. and 1:00 p.m. for all rats. The CPP apparatus (Med Associates Inc., Fairfax VT) consisted of three distinct compartments (white, black and neutral central gray) separated by removable doors. Locomotor activity and time spent in each compartment were monitored using infrared photobeams.

The CPP protocol followed a 14-day sequence: 1) preconditioning (day 1): The rats were allowed free access to the entire apparatus for 30 min. The preconditioning test showed a compartment bias for both cohorts 1 and 3 (Reich et al., 2019; Ryan et al., 2018); subsequently, Oxy was administered in the least preferred side during conditioning (i.e., biased CPP design). 2) CPP training (days 2-9): Rats underwent 4 training sessions. On the first day of each session, the rats were injected with Oxy (3 mg/kg, i.p.) and placed in one compartment (e.g., black) for 30 min [a time point within the 3–5 h half-life of Oxy (Ordonez Gallego et al., 2007)]. On the second day of each session, the rats were injected with Sal and placed in the other compartment (e.g., white) for 30 min. Control rats received Sal prior to both days of each of the four training sessions. 3) CPP test (day 14): Four days after last injection, the rats were placed in the neutral, central gray compartment and allowed free access to the entire apparatus with their behavior monitored for 30 min. Percent time in the Oxy-paired compartment was calculated by dividing the time spent in the Oxy-paired compartment over the time spent in both compartments. Preference score was calculated by subtracting the percent time in the Oxy-paired compartment during the pre-test from that of the post-test. Our prior studies (Reich et al., 2019; Ryan et al., 2018) reported that the naïve female and male rats used in cohort 1 both acquired Oxy CPP while CIS female rats, but not CIS male rats, used in cohort 3 acquired Oxy CPP.

### 2.5 Immunocytochemistry procedures

#### 2.5.1 Section preparation

CIS rats and their corresponding control rats (cohort 2) were euthanized 1 day after the last stress session (Mazid et al., 2016). Sal- and Oxy-CPP rats (cohorts 1 and 3) were euthanized immediately after the final training session (Reich et al., 2019; Ryan et al., 2018). Rats were deeply anesthetized either with sodium pentobarbital (150 kg/mg, i.p.; cohort 2) or with a mixture of ketamine (100 mg/kg) and xylazine (10 mg/kg; cohorts 1 and 3) and perfused through the ascending aorta sequentially with: 1) 10–15 ml 0.9% Sal, 2) 50 ml of 3.75% acrolein and 2% paraformaldehyde (PFA) in 0.1 M phosphate buffer (PB, pH = 7.4), and 3) 200 ml of 2% PFA in PB. Brains were removed from the skull, cut into coronal blocks (5 mm thick), and post-fixed in 2% PFA in PB for 30 min before being transferred into PB. Sections (40 μM thick) through the dorsal hippocampal [-3.5 to −4.2 mm from Bregma (Swanson, 1992)] were cut using a Vibratome (Leica Microsystems, Buffalo Grove, IL) and collected into 24-well plates containing PB. Sections were stored in a cryoprotectant solution (30% sucrose and 30% ethylene glycol in PB) at −20 °C. To ensure identical exposure to all reagents between groups, the sections were coded with hole punches and placed in single containers to be processed through all immunocytochemical procedures simultaneously (Pierce et al., 1999). Tissue from each cohort was processed as a single experiment. Sections were incubated in 1% sodium borohydride in PB for 30 min to neutralize reactive aldehydes (Milner et al., 2011) then rinsed 8–10 times in PB.

#### 2.5.2 Antibody characterization

**DOR:** A rabbit polyclonal antibody against amino acids 3–17 (LVPSARAELQSSPLV) in the N-terminus of the DOR protein (AB1560, Millipore, Temecula, CA) was used in this study. This antibody recognizes DOR1 in mouse, rat and human. An extensive description of previous characterization studies can be found in Mazid et al. (2016). Briefly, the DOR AB1560 has been characterized in Western blots of lysates from rat brains and in NG108-15 cells, which endogenously express DORs (Barg et al., 1984; Persson et al., 2005; Saland et al., 2005) and in preadsorption controls on tissue sections (Olive et al., 1997). Moreover, no detectable labeling of this antibody is seen in Western blots of COS-7 cells [see Supplemental Fig. 1 in (Williams et al., 2011b)], which do not endogenously express DORs (Kieffer et al., 1992) and in the dorsal raphe of DOR knockout mice [see Supplemental Fig. 1 in (Bie et al., 2010)]. Further, greater DOR-immunoreactivity (-ir) with AB1560 in interneurons compared to pyramidal cells in the rat hippocampus is consistent with DOR mRNA expression in this species (Mansour et al., 1994). Previous autoradiography experiments have described DOR binding sites in the rat hippocampus that corroborate the DOR-ir seen with this antibody (Crain et al., 1986; Gulya et al., 1986; Mansour et al., 1987; McLean et al., 1987).

The AB1560 antibody has been used in our previous light and electron microscopy studies (Commons and Milner, 1996; Mazid et al., 2016; Reich et al., 2019; Ryan et al., 2018; Williams and Milner, 2011; Williams et al., 2011b). DOR-ir in the rat hippocampus is sensitive to fixation, and thus yields the most intense labeling with 3.75% acrolein and 2% PFA fixed sections compared to 4% PFA fixed sections (Commons and Milner, 1997).

**GABA**: A rat polyclonal antiserum selective against GABA-glutaraldehyde-hemocyanin conjugates was provided courtesy of Dr. Andrew Towle. The specificity of this antiserum was confirmed using preadsorption with GABA-bovine serum albumin (BSA), where it was found to eliminate immunoreactivity. However, preadsorption with unconjugated GABA or BSA conjugated to glutamate, β-alanine or taurine did not abolish immunoreactivity (Lauder et al., 1986). The overall immunoreactivity of this antiserum is found to be consistent with that of other GABA-specific antisera (Lauder et al., 1986). This antibody has been used in previous light and electron microscopic studies (Drake and Milner, 1999; Mazid et al., 2016; Reich et al., 2019; Ryan et al., 2018).

### 2.6 Light microscopic immunocytochemistry

#### 2.6.1 Experimental procedure

To examine changes in the density of DOR-ir in the hippocampal CA1 region, sections were processed using previously described methods (Milner et al., 2011). Briefly, tissue sections were rinsed in 0.1 M Tris-buffered Sal (TS; pH = 7.6) and blocked in 0.5% BSA in TS for 30 min prior to incubation in rabbit anti-DOR antibody (1:5000) in 0.1% BSA and TS for 24 h at room temperature (25 °C) followed by an additional 24 h incubation at 4 °C. Sections then were incubated in a 1:400 dilution of donkey-anti-rabbit IgG (Jackson Immunoresearch Laboratories, Cat# 711-506-152, RRID:AB_2616595) for 30 min and rinsed in TS. Next, sections were incubated in avidin-biotin complex (ABC; Vectastain elite kit, Vector Laboratories, Burlingame, CA) at half the manufacturer's recommended dilution for 30 min, washed in TS, and reacted in 3,3′-diaminobenzidine (DAB; Sigma-Aldrich, St. Louis, MO) in 3% H_2_O_2_ in TS for 3.5 min. Tissue sections then were rinsed in PB and mounted from 0.05 M PB onto 1% gelatin-coated glass slides. The slides were dehydrated through an ascending series of ethanol concentrations and coverslipped from xylene using DPX mounting media (Sigma-Aldrich).

#### 2.6.2 Analysis: DOR levels

**Fig. 1:**
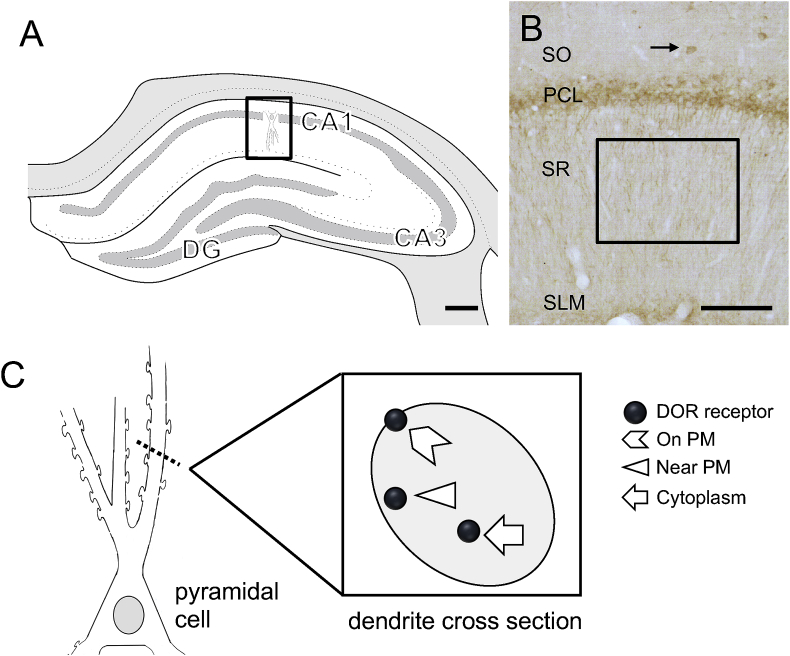
**Region of the rat hippocampus sampled for light and electron microscopy. A**. Schematic diagram of the rostral rat hippocampus showing the regions of CA1, CA3 and dentate gyrus (DG). CA1 (boxed region) was sampled for microscopy [modified from diagram 31 (−3.70 from bregma) in (Swanson, 1992)]. **B.** Representative light micrograph shows DOR-labeled soma are primarily located in the pyramidal cell layer (PCL) and in scattered interneurons (example arrow) within stratum oriens (SO). Boxed region indicates region of stratum radiatum (SR) sample for electron microscopy. **C.** Schematic diagram illustrates ultrastructural distribution of SIG particles in CA1 SR dendrite cross-section. SLM, stratum lacunosum-moleculare. Scale bar A: 0.5 mm; B: 100 μm.

A person blinded to experimental conditions performed the analyses. Densitometric quantification for DOR-ir within the CA1 region of the hippocampus (Fig. 1A) was done using previously described methods (Pierce et al., 2014; Williams and Milner, 2011; Williams et al., 2011b). Briefly, images of the hippocampus were captured at 4x on a Nikon Eclipse 80i microscope using a Micropublisher 5.0 digital camera (Q-imaging, BC, Canada) and IP Lab software (Scanalytics IPLab, RRID:SCR_002775). Average pixel density within regions of interest (ROI) was determined using ImageJ64 (ImageJ, RRID:SCR_003070) software. Stratum oriens (SO), pyramidal cell layer (PCL), near stratum radiatum (nSR), distal stratum radiatum (dSR), and stratum lacunosum-moleculare (SLM) were measured. To control for variations in overall illumination levels between images and to compensate for background staining, the pixel density of glass was subtracted from ROI measurements. Prior studies corroborate the accuracy of this technique by showing a strong linear correlation between average pixel density and neutral density values of gelatin filters with defined transmittances ranging from 1 to 80% (Eastman Kodak Co.,) (Auchus and Pickel, 1992; Pierce et al., 1999, 2014).

### 2.7 Dual labeling electron microscopic immunocytochemistry

#### 2.7.1 Experimental procedure

Tissue sections were dual labeled for DOR and GABA, as described in our prior studies (Mazid et al., 2016; Milner et al., 2013). Briefly, tissue sections were rinsed in TS and blocked in 0.5% BSA in TS for 30 min. Sections were placed in a cocktail of anti-DOR (1:5000) and anti-GABA (1:1000) in 0.1% BSA in TS and placed on a shaker for 24 h at room temperature and then for 4 days at 4 °C.

Next, sections were processed for peroxidase labeling for GABA (3 min in DAB) as described above, except that biotinylated donkey anti-rat IgG (GABA; Jackson Immunoresearch Laboratories, Cat# 712-065-150, RRID:AB_2340646) was used as a secondary antibody. Sections then were washed in TS and incubated in donkey anti-rabbit IgG (DOR; Jackson Immunoresearch Laboratories, Cat#711-506-152, RRID:AB_2616595) conjugated to 1 nm gold particles [diluted 1:50; Electron Microscopy Sciences (EMS), Cat# 810.311, RRID:AB_2629850] in 0.01% gelatin and 0.08% BSA in 0.01 M phosphate-buffered Sal (PBS) at 4 °C overnight. Tissue sections then were rinsed in PBS, postfixed in 2% glutaraldehyde in PBS for 10 min, and rinsed in PBS followed by 0.2 M sodium citrate buffer (pH = 7.4). IgG conjugated gold particles were enhanced by undergoing a reaction for 6–7 min with a silver solution [cohort 2: RPN Silver Enhance kit, GE Healthcare, Waukeska, WI (discontinued); cohorts 1 & 3: SEKL15 Silver enhancement kit, Prod No. 15718 Ted Pella Inc.].

Tissue sections were fixed in 2% osmium tetroxide for 1 h followed by washes of PB and dehydration via increasing concentrations of ethanol and propylene oxide prior to embedding in EMbed 812 (EMS, #14120). Ultrathin sections (approximately 70–72 nm thick) through the CA1 region were cut on a Leica UCT ultramicrotome and collected on 400 mesh thin-bar copper grids (EMS, T400-Cu), which were then counterstained with UranyLess™ (EMS, #22409) and lead citrate (EMS, #22410).

#### 2.7.2 Identification of EM profiles

Tissue sections containing the hippocampal CA1 region were examined on a CM10 (cohort 2) or Tecnai Biotwin (cohorts 1 and 3) transmission electron microscope (FEI, Hillsboro, OR). Images were captured at a magnification of 13000x (CM10) or 13500x (Tecnai) and profiles identified based on standard morphology (Peters et al., 1991). CA1 pyramidal cell dendrites in SR often contained microtubules, agranular reticulum, and mitochondria that run parallel to the axis. These dendrites frequently exhibited spines that were post-synaptic to axon terminals containing numerous small synaptic vesicles forming asymmetric synapses and/or spiny protuberances that gave their plasmalemma a slightly erose appearance. Immunoperoxidase precipitate for GABA identified interneuron dendrites which lacked spines and often received synaptic contacts from numerous terminals. Peroxidase labeling was diffuse and granular whereas silver intensified immunogold (SIG) labeling for DOR appeared as black electron-dense particles (Milner et al., 2011). Data were collected and analyzed by investigators who were blind to experimental condition. Experimental condition was unblinded after figures were generated.

Analysis 1: DOR-SIG labeling in pyramidal cell dendrites in CA1. Micrographs were taken of dendrites labeled with DOR-SIG only in the CA1 SR (~50 μm away from the pyramidal cell layer) (Fig. 1B). The criteria for dendritic selection were: 1) profiles that lacked GABA immunoreactivity, 2) contained at least one spine or spine-like protuberance and 3) had an average diameter larger than 0.4 μm. Photographs were taken of 50 random DOR-SIG labeled dendritic profiles for each animal to ensure that the sampling of different dendritic regions was unbiased between samples. The location of the micrographs collected were noted on a low magnification photograph of the block surface so that samples were not double counted. In most cases, one thin section per block yielded the required number of dendrites but rarely dendritic images from a non-overlapping region of a second section were collected. Three rats per experimental group were analyzed yielding 150 dendrites per group for the statistical analysis.

Next, Microcomputer Imaging Device software (MCID Analysis, RRID:SCR_014278) was used to determine perimeter (i.e., plasma membrane), area, average diameter, and major and minor axis lengths for all labeled dendrites. Average diameter measures were used to classify dendrites as large (>1.0 μm) or small (between 0.4 and 1.0 μm). The location of DOR-SIG particles (on plasmalemma, near plasmalemma or within the cytoplasm) for each dendritic profile was noted during the MCID analysis. Once collected, the density and partitioning ratio of DOR-SIG particles within the dendrites were calculated. The density of DOR-SIG particles was analyzed in distinct dendritic compartments: 1) the number of plasma membrane DOR-SIG particles on the dendrite perimeter (On PM:μm); 2) the number of near (adjacent but not touching) plasma membrane DOR-SIG particles per perimeter (Near:μm); 3) the number of cytoplasmic DOR-SIG particles per cross-sectional area (Cyto:μm^2^); and 4) the total number of DOR-SIG particles (sum of on PM, near PM and cytoplasmic) in a dendritic profile/unit area (Total:μm^2^). Partitioning ratio, which is the proportion of DOR-SIG particles in a particular subcellular compartment (e.g., plasma membrane or cytoplasm) divided by the total number of SIG particles, also was determined.

The location of DOR-SIG particles within dendrites has functional significance (Fig. 1C). Receptors on the plasma membrane labeled by SIGs identify receptor-binding sites (Boudin et al., 1998). Near plasma membrane receptors constitute a pool from which receptors can be added or removed from the plasma membrane. Receptors in the cytoplasm are either stored during transfer to or from the soma or another cellular compartment, or the receptors are being degraded or recycled (Fernandez-Monreal et al., 2012; Pierce et al., 2009). The internalization of epitope-tagged MORs in the nucleus accumbens following morphine administration supports the functionality of receptor trafficking using SIG (Haberstock-Debic et al., 2003).

Analysis 2: DOR-SIG labeling in dendritic spines in CA1.

From the micrographs of CA1 SR collected in Analysis 1, 100 spine profiles per rat were randomly identified. Spines were included if contacted by a terminal forming an asymmetric synapse and categorized as labeled (with at least one DOR-SIG particle) or unlabeled. DOR-SIG particles in labeled spines were classified as in the synapse, on the plasma membrane, or in the cytoplasm.

### 2.8 Statistical analysis and figure preparation

Sex (female vs. male) and treatment (cohort 1: Sal vs Oxy CPP; cohort 2: unstressed control vs. CIS; cohort 3: CIS Sal vs CIS Oxy CPP) were the independent variables in this study. The quantitative dual labeling EM methods used in this study are planned to compare relative changes in the subcellular distribution of proteins within different sized dendrites following experimental manipulations (Milner et al., 2011). For this, the distribution of DORs in dendritic profiles of different sizes, rather than number of cells or dendrites per rat, is analyzed. Implicit in this analysis are corrections for errors related to spatial location as dendritic profiles are collected from a single plane within each section. In our studies, dendritic profile perimeter, cross-sectional area, and average diameter are measured and then used to determine SIG particle density for each dendritic compartment (e.g., cyto/μm^2^) to correct for any size-related differences. To analyze the partitioning ratio of SIG particles within a dendrite, the number of SIG particles in each compartment was divided by the total number of SIG particles in the dendrite (e.g., Cyto/total).

We (Znamensky et al., 2003) previously determined that 50 dendritic profiles per block were sufficient to make quantitative comparisons of the subcellular distribution of proteins between groups. We (Znamensky et al., 2003) also found that increasing dendritic number to 75 or more per rat did not significantly alter the results.

Data are expressed as means ± SEM. All statistical analyses were conducted on JMP 12 Pro software (JMP, RRID:SCR_014242) and significance was set to an alpha <0.05. Light microscopic optical density sample comparisons and comparisons in DOR-SIG distributions in the Sal-injected female and male unstressed and CIS rats were determined through one-way ANOVA or two tailed *t*-test with a Welch correction for samples with unequal variances (as determined by Levene's test). Comparisons between sex and treatment (unstressed vs. CIS, Oxy vs Sal CPP) were analyzed for main effects with two-way ANOVAs, using Tukey's HSD post-hoc analyses for simple effects.

As the EM findings reflect a statistical analysis of numerous micrographs, it is difficult to select a single micrograph that reflects all of the DOR distribution changes seen after a manipulation. Several issues were carefully considered when selecting the representative micrographs. Most importantly, the micrographs were selected so that the distribution of the DOR-SIG particles in the four groups reflected the data in the graphs without contradicting it (e.g., greater total SIG particles in a particular group than shown in the graphs). Moreover, micrographs were selected based on morphological preservation and near equivalence of size. Lastly, the micrographs were selected to illustrate density changes, not partitioning ratio changes, in DOR-SIG particles.

Images were adjusted for size, sharpness, and contrast in Adobe Photoshop 9.0 (Adobe Photoshop, RRID:SCR_014199) prior to importing them into Microsoft PowerPoint 2010, where final adjustments to sharpness, brightness, and contrast were made, none of which significantly altered the appearance of the initial raw image. These adjustments were made to achieve a uniform appearance between electron micrographs. Graphs were generated using Prism 8 software (Graphpad Prism, RRID:SCR_002798).

## 3 Results

### 3.1 COHORT 1. Oxy CPP in naïve rats

#### 3.1.1 By light microscopy, DOR levels in CA1 were similar in all four groups

By light microscopy, labeling pattern of DOR-immunoreactivity (ir) in the CA1 of female and male rats was consistent with previous findings (Commons and Milner, 1997). DOR-labeling was found in interneurons in SO and in cell bodies in the pyramidal cell layer (PCL) as well as dendrites in SR (Williams et al., 2011b). A representative image showing DOR-ir in CA1 is shown in Fig. 1B. Light microscopic densitometric analysis of DOR-ir in all lamina of CA1 showed no differences between Sal- and Oxy-females and males (not shown).

#### 3.1.2 Sal-females have greater DOR densities in CA1 dendrites compared to males

**Fig. 2:**
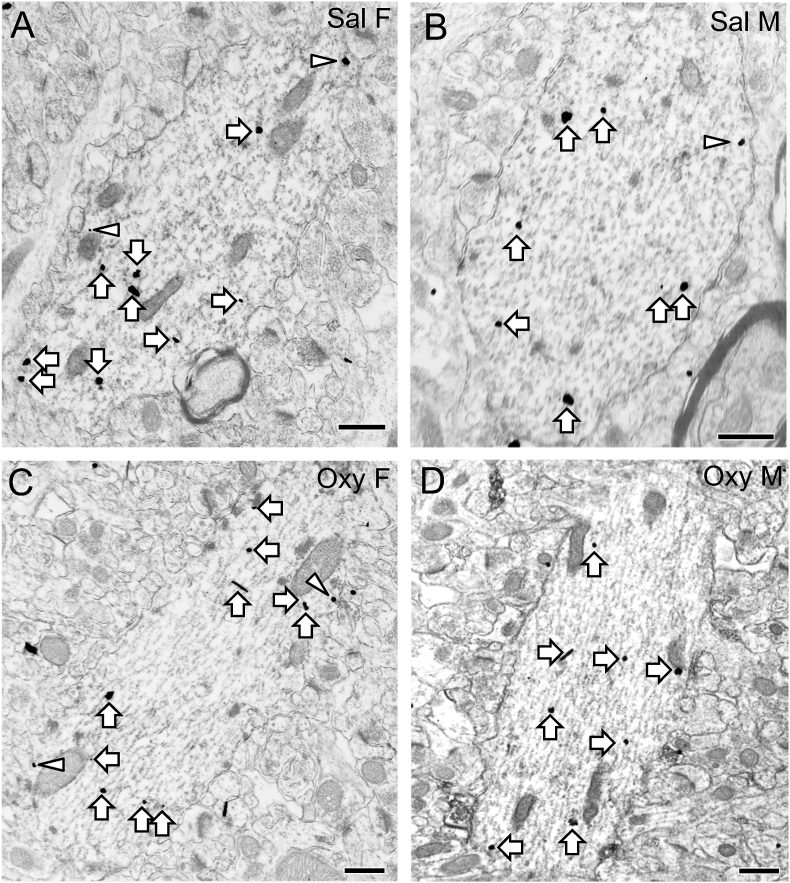
**Representative electron micrographs of DOR-SIG particles in CA1 pyramidal cell dendrites from naïve Sal- and Oxy-female and male rats**. Electron micrographs show the distribution of DOR-SIG particles within CA1 pyramidal cell dendrites from a Sal-female (**A**), a Sal-male (**B**), an Oxy-female (**C**), and an Oxy-male (**D**) rat. Examples of near plasmalemmal (triangle) and cytoplasmic (arrow) DOR-SIG particles in dendrites are shown. Scale bar: 500 nm.

**Fig. 3:**
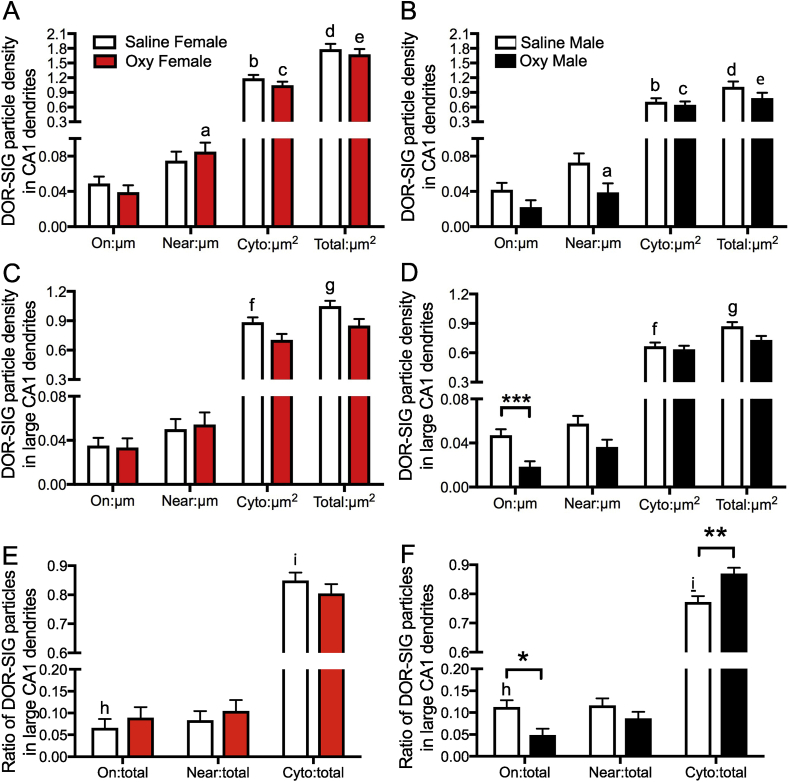
**Sex differences in the distribution of DOR-SIG particles in CA1 dendrites in Sal- and Oxy-female and male rats. A,B**. Sal-females compared to Sal-males have a greater density of DOR-SIG particles in the cytoplasm and in total in CA1 pyramidal cell dendrites. Oxy-females compared to Oxy-males have a greater density of DOR-SIG particles near the plasmalemma, in the cytoplasm, and in total in CA1 pyramidal cell dendrites. **C–F.** When dendrites are separated based on size, Sal-females compared to Sal-males have a greater density of DOR-SIG particles in the cytoplasm and in total in large CA1 dendrites (**C,D**). Sal-females compared to Sal-males have a lower ratio of plasmalemmal DOR-SIG particles and a greater ratio of cytoplasmic DOR-SIG particles in large CA1 dendrites (**E,F**). Oxy-males compared to Sal-males have a lower density (**D**) and partitioning ratio (**F**) of plasmalemmal DOR-SIG particles and a greater ratio of cytoplasmic DOR-SIG particles (**F**) in large CA1 dendrites. ^e^p < 0.0001, ***p, ^b-d^p < 0.001,**p < 0.01, ^a,f^p < 0.01, *p < 0.05, ^g,i^p < 0.05, ^h^p = 0.058, N = 3 rats per group; n = 50 dendrites per rat.

Representative electron micrographs showing DOR-SIG labeling in CA1 pyramidal cell dendrites for all four groups are shown in Fig. 2A–D. Sal rats in Cohort 1 showed significant sex differences in the subcellular distribution of DORs in CA1 dendrites. Compared to Sal-males, Sal-females showed a greater density of DOR-SIG particles in the cytoplasm (F_1,195_ = 18.2400; p < 0.0001) and in total (F_1,185_ = 20.7578; p < 0.0001) in CA1 dendrites (Fig. 2, Fig. 3A,B). When dendrites were separated by size, the same finding was observed both in cytoplasmic and total density of DOR-SIG particles in large (F_1,110_ = 8.6896; p = 0.0039, F_1,109_ = 4.4222; p = 0.0378, respectively; Fig. 3C,D) and small (F_1,117_ = 7.4023; p = 0.0075, F_1,118_ = 7.9734; p = 0.0056, respectively; not shown) dendrites.

#### 3.1.3 Both Sal- and Oxy-females show increased DOR density in CA1 dendrites compared to males

Two-way ANOVA showed significant main effects of sex on DOR-SIG density near the plasma membrane (F_3,590_ = 5.5885; p = 0.0184), in the cytoplasm (F_3,590_ = 37.5915; p < 0.0001), and in total (F_3,590_ = 57.5142; p < 0.0001) in CA1 dendrites. A sex by treatment interaction was shown for DOR-SIG density near the plasma membrane (F_3,590_ = 4.6427; p = 0.0316) of CA1 dendrites. Post-hoc analysis revealed a significantly greater density of DOR-SIG particles near the plasma membrane (p = 0.0079), in the cytoplasm (p = 0.0005), and in total (p < 0.0001) in dendrites in Oxy-females compared to Oxy-males (Fig. 2, Fig. 3A,B).

When dendrites were divided based on size, there were significant main effects of treatment (Sal vs Oxy) in density of DOR-SIG particles on the plasma membrane (F_3,342_ = 5.3759; p = 0.0210), in the cytoplasm (F_3,342_ = 5.0118; p = 0.0258), and in total (F_3,342_ = 10.4742; p = 0.0013) in large dendrites. There was a significant main effect of sex on DOR-SIG density in the cytoplasm (F_3,342_ = 9.3281; p = 0.0024, F_3,244_ = 8.2261; p = 0.0045, respectively) and in total (F_3,342_ = 8.1842; p = 0.0045, F_3,244_ = 10.6371; p = 0.0013, respectively) in large and small dendrites. A sex by treatment interaction was shown for DOR-SIG density on the plasma membrane (F_3,342_ = 4.1724; p = 0.0419) of large dendrites. Post-hoc analysis showed that Oxy-males had decreased density of DOR-SIG particles on the plasma membrane of large CA1 dendrites (p = 0.0006; Fig. 3D) compared to Sal-males. No change in DOR-SIG particle density in large dendrites was seen in Oxy-females compared to Sal-females (Fig. 3C).

#### 3.1.4 Sal-males have a higher ratio of plasmalemmal DORs but this is reversed after Oxy-CPP

Two-way ANOVA showed a significant main effect of sex in the partitioning ratios of DOR-SIG particles near the plasma membrane (F_3,590_ = 6.2813; p = 0.0125) and in the cytoplasm (F_3,590_ = 5.6644; p = 0.0176) of CA1 dendrites. A sex by treatment interaction was shown for the partitioning ratio of DOR-SIG particles near the plasma membrane (F_3,590_ = 9.1374; p = 0.0026) and in the cytoplasm (F_3,590_ = 10.9471; p = 0.0010) of CA1 dendrites. Post-hoc analysis showed that the proportion of DOR-SIG particles in Oxy-females was greater near the plasma membrane (p = 0.0006) and in the cytoplasm (p = 0.0004) of CA1 dendrites compared to Oxy-males (Figs. 2C,D, 3A,B). The ratio of cytoplasmic to total DOR-SIG particles increased in Oxy-males compared to Sal-males (p = 0.0184; Fig. 2, Fig. 3).

When separated by size (large vs small), a sex by treatment interaction was shown for DOR-SIG partitioning ratio on the plasma membrane (F_3,342_ = 5.4913; p = 0.0197) and in the cytoplasm of large dendrites (F_3,342_ = 7.9379; p = 0.0051) and near the plasma membrane (F_3,244_ = 4.6451; p = 0.0321) of small dendrites. Post-hoc analysis showed that the proportion of DOR-SIG particles in Sal-females compared to Sal-males tended to decrease on the plasmalemma (p = 0.0578) and increased in the cytoplasm (p = 0.0159) of large dendrites (Fig. 3E,F). Oxy-males had a decreased ratio of DOR-SIG particles on the plasma membrane (p = 0.0124) and an increased ratio of DOR-SIG particles in the cytoplasm (p = 0.0034) of large dendrites compared to Sal-males (Fig. 2, Fig. 3F).

#### 3.1.5 DOR-labeled dendritic spines increase in Oxy-males

**Fig. 4:**
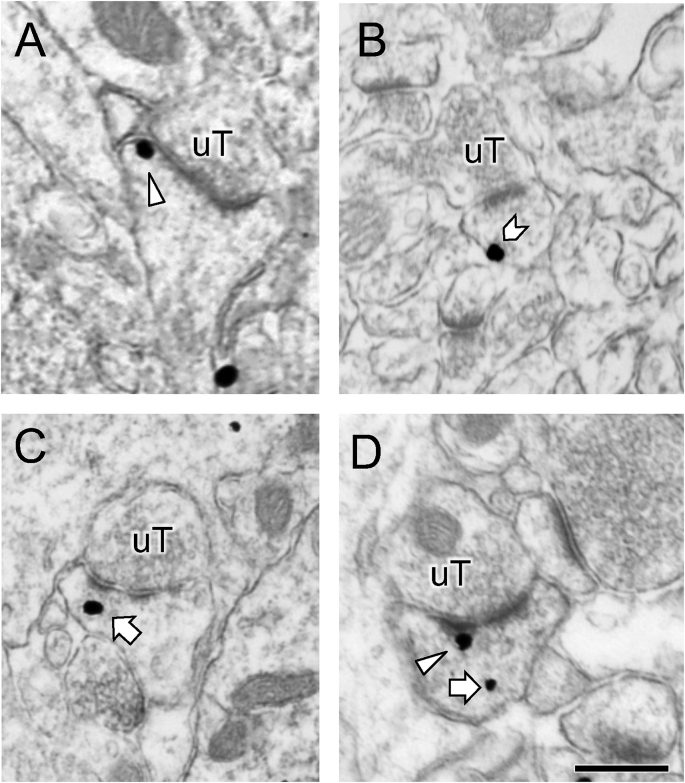
**Examples of DOR-labeled spines in CA1**. Usually one DOR-SIG particles was found in a single dendritic spine. DOR-SIGs were found near the synapse (triangle; **A**), on the plasma membrane (chevron; **B**) or in the cytoplasm (arrow; **C**). Sometimes, more than one DOR-SIG particle was detected in a dendrtic spine (**D**). Spines were contacted by unlabeled terminals (uT). Images taken from control female rats (cohort 2). Scale bar: 500 nm.

Consistent with prior studies (Williams et al., 2011b), DOR-SIG particles were found in dendritic spines in CA1 SR (Fig. 4). The percent of DOR labeled spines and distribution of DORs within the spines is shown in Table 1. In a single spine, usually only one SIG particle was detected (Fig. 4A–C), although sometimes two SIG particles were observed (Fig. 4D). As sample sizes were too low to perform statistical analyses for DOR labeling in the subcompartments of CA1 dendritic spines, qualitative descriptions are presented for this data. The percent of DOR-labeled dendritic spines is not significantly different in Sal-females compared to Sal-males. However, the majority of DOR-SIG particles were in the cytoplasm (Fig. 4C) in Sal-males and on the plasma membrane (Fig. 4B) in Sal-females. Following CPP, the percent of DOR-labeled spines significantly increased (p = 0.048) in Oxy-males compared to Sal-males. Moreover, the number of DOR-SIG particles on the plasma membrane increased in Oxy-males but decreased in Oxy-females (Table 1).

**Table 1:** DOR-labeled spines in CA1.

Group	% ± SEM labeled spines	Total labeled spines	Location+		
			synapse	membrane	cytoplasm
**OXY CPP**					
Sal Male	6.0 ± 1*	18	1	3	14
Oxy Male	10.67 ± 1.3*	32	2	10	20
Sal Female	8 ± 0.6	24	3	14	7
Oxy Female	8.3 ± 1.5	25	2	8	16

**CIS**					
Con Male	7.7 ± 2.2	23	3	5	16
CIS Male	6.7 ± 2.9	20	3	6	11
Con Female	5.4 ± 0.8	18	10	2	6
CIS Female	8.8 ± 2.6	27	7	2	18

**CIS OXY CPP**					
CIS Sal Male	2.67 ± 0.3***	8	1	2	6
CIS Oxy Male	3.0 ± 1.5	9	1	1	7
CIS Sal Female	7.67 ± 0.3***	23	5	10	9
CIS Oxy Female	7.33 ± 0.3	22	3	7	13

+Some spines had more than one DOR-SIG particle.

*p = 0.048.

***p = 0.0004.

### 3.2 COHORT 2. unstressed vs CIS rats

#### 3.2.1 By light microscopy, DOR levels in CA1 were similar in all four groups

At the light microscope level, control and CIS females and males had comparable densities of DOR-ir in all lamina of CA1 (not shown). This observation is consistent with our previous study analyzing the density of DOR in CA3 from these same animals (Mazid et al., 2016).

#### 3.2.2 Unstressed females have more DORs near the plasma membrane compared to males

**Fig. 5:**
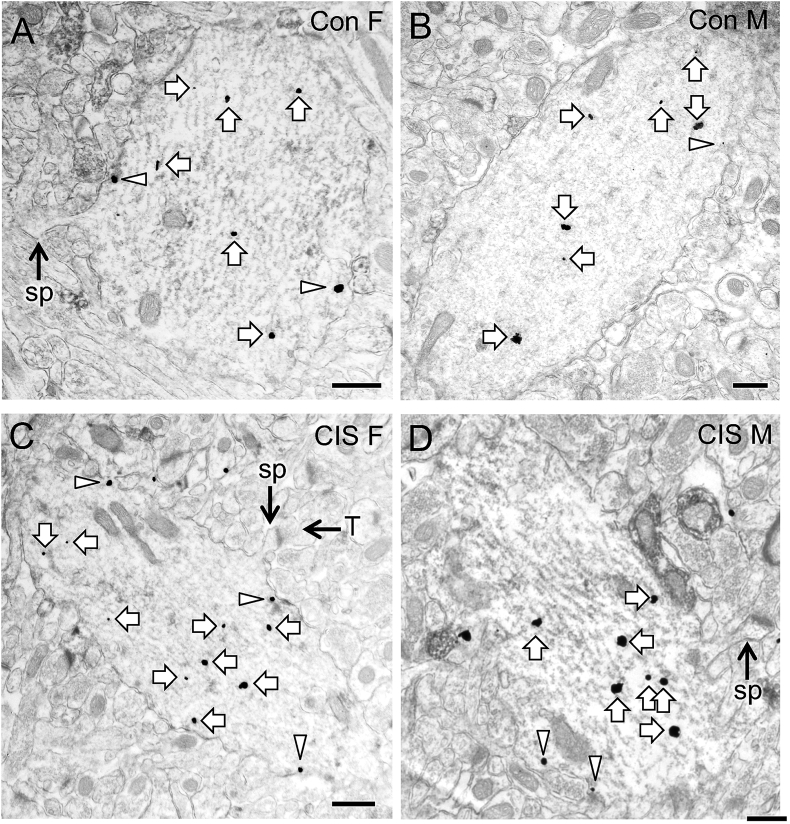
**Representative electron micrographs of DOR-SIG particles in CA1 pyramidal cell dendrites from control and CIS female and male rats**. Electron micrographs show the distribution of DOR-SIG particles within CA1 pyramidal cell dendrites from a control female (**A**), a control male (**B**), a CIS female (**C**), and a CIS male (**D**) rat. Examples of near plasmalemmal (triangle) and cytoplasmic (arrow) DOR-SIG particles in dendrites are shown. CA1 dendrites often have spines (sp) that are contacted by terminals (example A, C and D). Scale bar: 500 nm.

**Fig. 6:**
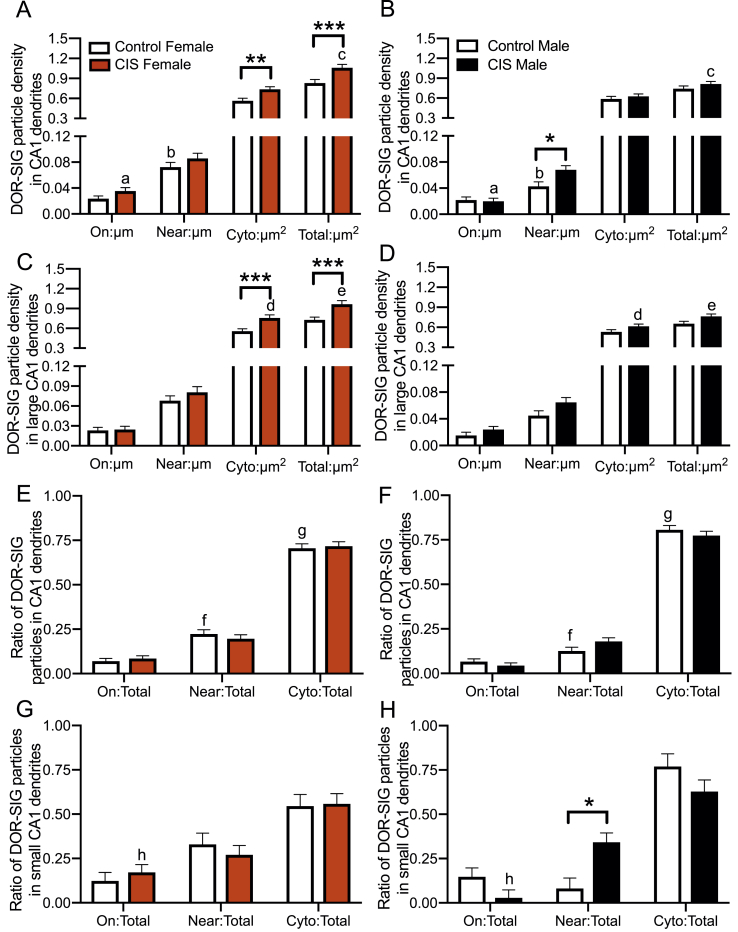
**Sex differences in the distribution of DOR-SIG particles in CA1 dendrites in control and CIS female and male rats. A,B**. Control females have a higher density of DOR-SIG particles near the plasma membrane of CA1 pyramidal cell dendrites compared to control males. Following CIS, females have an increased density of DOR-SIG particles in the cytoplasm and in total while males have an increased density of near plasmalemmal DOR-SIG particles in CA1 dendrites. CIS females compared to CIS males have a higher density of plasma membrane and total DOR-SIG particles on CA1 dendrites. **C,D.** In large CA1 dendrites, CIS females have increased cytoplasmic and total DOR-SIG particle density compared to control females and CIS males. **E,F.** Control females compared to control males have a greater ratio of near plasmalemmal DOR-SIG particles and a lower ratio of cytoplasmic DOR-SIG particles in CA1 pyramidal cell dendrites. **G,H.** In small CA1 pyramidal cell dendrites, CIS males have an increased ratio of near plasmalemmal DOR-SIG particles compared to control males. CIS females compares to CIS males have an increased ratio of plasma membrane DOR-SIG particles in small CA1 dendrites. ***p < 0.001, **p < 0.01, *p < 0.05, ^a,c,g^p < 0.05, ^b,f^p < 0.01, ^d^p = 0.051, ^e^p < 0.05, ^h^p = 0.06. N = 3 rats per group; n = 50 dendrites per rat.

Electron micrographs of CA1 pyramidal cell dendrites showing examples of DOR-SIG particles on the plasma membrane, near the plasma membrane, and in the cytoplasm from naïve and CIS rats are shown in Fig. 5A–D. Compared to unstressed males, unstressed females had a higher density of DOR-SIG particles near the plasma membrane (p = 0.0130) in CA1 dendrites (Fig. 5, Fig. 6A,B).

#### 3.2.3 CIS increases DOR-SIG particles in both sexes, albeit in differing locations

Two-way ANOVA showed a significant main effect of sex on the density of DOR-SIG particles on the plasma membrane (F_3,597_ = 2.5828; p = 0.0489), near the plasma membrane (F_3,597_ = 6.8256; p = 0.0005), and in total (F_3,597_ = 9.9297; p = 0.0005) in CA1 dendrites. A main effect for treatment (unstressed vs. CIS) was observed in the DOR-SIG particle density near the plasma membrane (F_3,597_ = 6.8256; p = 0.0050), in the cytoplasm (F_3,597_ = 4.2301; p = 0.0043), and in total (F_3,597_ = 9.9297; p = 0.0003). Sex by treatment interaction was significant for total DOR-SIG particle density (F_3,597_ = 9.9297; p = 0.0365) in CA1 dendrites.

Post-hoc analysis revealed that CIS females had a significantly greater density of DOR-SIG particles in the cytoplasm (p = 0.0057) and in total (p = 0.0003) compared to unstressed females in CA1 dendrites (Fig. 5, Fig. 6A). CIS males had a significantly greater density of DOR-SIG particles near the plasma membrane (p = 0.0477) compared to unstressed males in CA1 dendrites (Fig. 5, Fig. 6B). CIS females had a higher density of DOR-SIG particles on the plasma membrane (p = 0.0565) and in total (p = 0.0005) in CA1 dendrites compared to CIS males (Fig. 5, Fig. 6A,B).

When divided by size (large vs small), significant main effects of sex were found in the density of DOR-SIG particles near the plasma membrane (F_3,448_ = 3.9717; p = 0.0079), in the cytoplasm (F_3,448_ = 8.0319; p = 0.0151), and in total (F_3,448_ = 10.3928; p = 0.0006) in large dendrites and near the plasma membrane (F_3,145_ = 2.5599; p = 0.0394) in small dendrites. Significant main effects for treatment were found in DOR-SIG particle density near the plasma membrane (F_3,448_ = 3.9717; p = 0.0312), in the cytoplasm (F_3,448_ = 8.0319; p < 0.0001), and in total (F_3,448_ = 10.3928; p < 0.0001) in large dendrites. The sex by treatment interaction for the DOR-SIG particle density was significant on the plasma membrane (F_3,145_ = 4.9052; p = 0.0006) in small dendrites.

Post-hoc analysis showed that CIS females had a higher DOR-SIG particle density in the cytoplasm (p = 0.0005) and in total (p = 0.0004) in large CA1 dendrites compared to unstressed females (Fig. 6C). Large dendrites in CIS females also had a higher density of DOR-SIG particles in the cytoplasm (p = 0.0194) and in total (p = 0.0026) compared to CIS males (Fig. 6C,D). CIS females also had higher DOR-SIG particle density on the plasma membrane (p = 0.0032) of small dendrites compared to CIS males (not shown).

#### 3.2.4 In males, the proportion of DORs near the plasma membrane increases following CIS

Two-way ANOVA showed a significant main effect of sex on the partitioning ratio of DOR-SIG particles near the plasma membrane (F_3,597_ = 3.6561; p = 0.0078) and in the cytoplasm (F_3,597_ = 4.0327; p = 0.0009) in CA1 dendrites. Post-hoc analysis showed that unstressed females compared to unstressed males had a greater DOR-SIG particle ratio near the plasma membrane (p = 0.0078; Fig. 6E,F) and a lower DOR-SIG particle ratio in the cytoplasm (p = 0.0151) of CA1 dendrites (Fig. 6E,F).

When divided by size (large vs small), significant main effects of sex were found in the partitioning ratio of DOR-SIG particles near the plasma membrane (F_3,448_ = 1.5793; p = 0.0471) in large dendrites and in the cytoplasm (F_3,145_ = 2.3569; p = 0.0220) in small dendrites. The sex by treatment interaction for the partitioning ratio of DOR-SIG particles was significant near the plasma membrane (F_3,145_ = 3.6782; p = 0.0066) in small CA1 dendrites.

Post-hoc analysis showed that CIS males had a higher proportion of DOR-SIG particles near the plasma membrane (p = 0.0233) in small dendrites compared to unstressed males (Fig. 6H). Compared to CIS males, CIS females had a higher proportion of DOR-SIG particles on the plasma membrane in small dendrites which approached significance (p = 0.0661; Fig. 6G,H).

#### 3.2.5 In females, DORs redistribute from the synapse to cytoplasm in DOR-labeled dendritic spines following CIS

The percent of DOR-labeled spines and distribution of DORs within the spines is shown in Table 1. The percent of DOR-labeled spines was not significantly different between the four groups. Of the labeled spines, DOR-SIG particles were primarily found in the cytoplasm (Fig. 4C) for control and CIS males as well as CIS females (Table 1). In contrast, DOR-SIG particles were primarily found on the synapse of control females.

### 3.3 COHORT 3. Oxy CPP in CIS rats

#### 3.3.1 By light microscopy, DOR levels in CA1 decrease in CIS females following Oxy-CPP

**Fig. 7:**
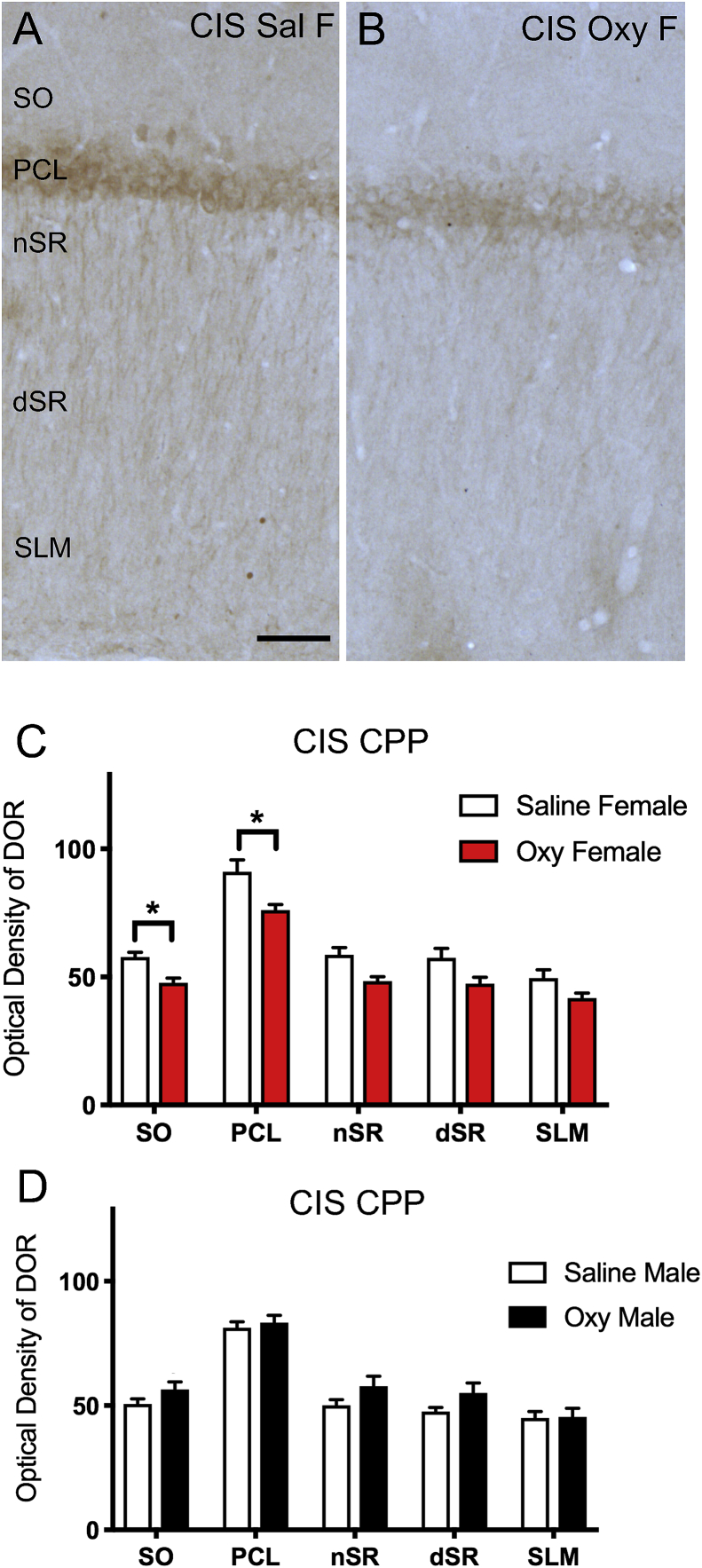
**DOR levels in CA1 are reduced in CIS Oxy-females**. A,B. Low magnification light photomicrographs of the rat CA1 show DOR-immunoreactivity in stratum oriens (SO), pyramidal cell layer (PCL), near stratum radiatum (nSR), distal stratum radiatum (dSR), and stratum lacunosum-moleculare (SLM) of the CA1 in a CIS Sal female (**A**) and a CIS Oxy-female (**B**) rat. Scale bar: 100 μm. **C.** CIS Oxy females compared to CIS Sal females have a lower density of DOR-immunoreactivity in the SO and PCL in CA1. **D.** No differences were observed in DOR-immunoreactivity in CA1 between CIS Sal- and Oxy-males. *^,a^ p < 0.05.

At the light microscope level, there were no differences in the densities of DOR-ir in any CA1 lamina between CIS Sal-females and CIS Sal-males (Fig. 7C,D). However, CIS Sal-females had a greater density of DOR-ir than CIS Oxy-females in the SO (F_3,20_ = 2.3915; p = 0.0338) and PCL (F_3,20_ = 2.6624, p = 0.013) of CA1 (Fig. 7A–C). No differences were seen in the levels of DOR-ir between CIS Sal- and Oxy-males in any lamina (Fig. 7D).

#### 3.3.2 The DOR density on the plasma membrane of large CA1 dendrites is greater in CIS Sal-female rats compared to CIS Sal-males

**Fig. 8:**
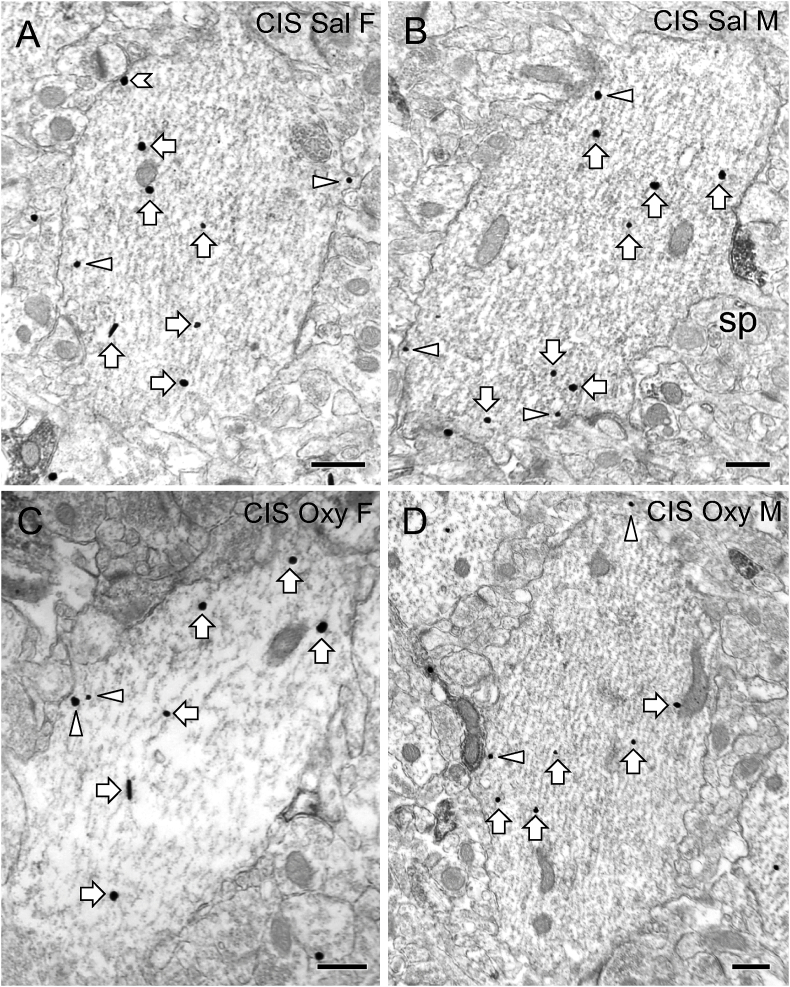
**Representative electron micrographs of delta opioid receptor (DOR) silver-intensified gold (SIG) particles in CA1 pyramidal cell dendrites from CIS Sal- and Oxy-female and male rats**. Electron micrographs show the distribution of DOR-SIG particles within CA1 pyramidal cell dendrites from a CIS Sal-female (**A**), a CIS Sal-male (**B**), a CIS Oxy-female (**C**), and a CIS Oxy-male (**D**) rat. Examples of on the plasmalemma (chevron), near plasmalemmal (triangle) and cytoplasmic (arrow) DOR-SIG particles in dendrites are shown. A dendritic spine (sp) is contacting a CA1 dendrite (example B). Scale bar: 500 nm.

**Fig. 9:**
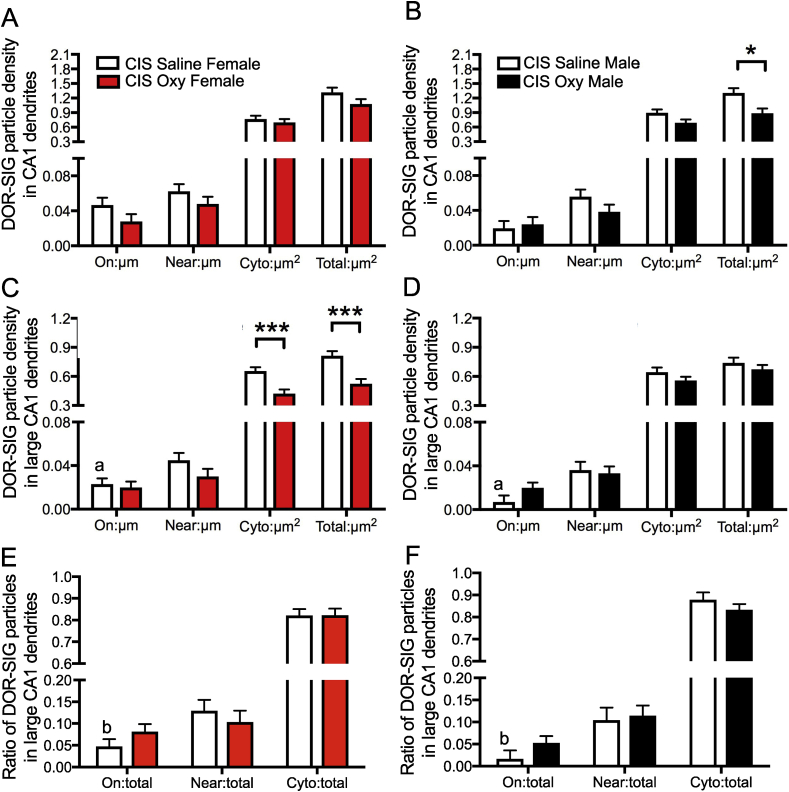
**Sex differences in the distribution of DOR-SIG particles in CA1 dendrites in CIS Sal- and Oxy-female and male rats**. A. No differences are observed in density of DOR-SIG particles in CA1 pyramidal cell dendrites between CIS Sal- and Oxy-females. CIS Sal-males and CIS Sal-females had similar densities of DOR-SIG particles in CA1 dendrites. **B.** CIS Oxy-males compared to CIS Sal-males have a lower density of DOR-SIG particles in total CA1 dendrites. **C–F.** When dendrites are separated based on size, CIS Sal-females compared to CIS Sal-males have a greater density (**C,D**) and partitioning ratio (**E,F**) of DOR-SIG particles on the plasma membrane in large CA1 dendrites. CIS Oxy-females compared to CIS Sal-females have a lower density of DOR-SIG particles in the cytoplasm and in total in large CA1 dendrites (**C**). No differences are observed in the density (**D**) or partitioning ratio (**F**) of DOR-SIG particles in large CA1 pyramidal cell dendrites between CIS Sal- and Oxy-males. ***p < 0.001, *p, ^a,b^p < 0.05, N = 3 rats per group; n = 50 dendrites per rat.

Representative electron micrographs showing DOR-SIG particles in CA1 dendrites from the four groups in the CIS CPP cohort are shown in Fig. 8A-D. Following CIS, Sal-rats showed no significant sex differences in the density of DOR-SIG particles in any compartment in CA1 dendrites (Fig. 9A,B). However, when dendrites were separated based on size, CIS Sal-females compared to CIS Sal-males had increased density of DOR-SIG particles on the plasma membrane (F_1,154_ = 5.69; p = 0.0185) in large CA1 dendrites (Fig. 9C,D).

#### 3.3.3 Few differences in DOR density are observed in CIS Oxy-females and CIS Oxy-males

Two-way ANOVA showed significant main effects of treatment on the density of cytoplasmic (F_3,594_ = 4.0198; p = 0.0454) and total (F_3,594_ = 10.8431; p = 0.0011) DOR-SIG particles in CA1 dendrites. Post-hoc analysis revealed a significantly decreased density of total DOR-SIG particles in dendrites in CIS Oxy-males compared to CIS Sal-males (p = 0.0157; Fig. 8, Fig. 9B).

When dendrites were divided based on size (large vs small), a significant main effect of treatment was observed in the density of cytoplasmic (F_3,334_ = 14.3829; p = 0.0002) and total (F_3,334_ = 13.2386; p = 0.0003) DOR-SIG particles of large dendrites (F_3,334_ = 4.1734; p = 0.0418). A sex by treatment interaction was significant (F_3,334_ = 5.2720; p = 0.0223) for the density of total DOR-SIG particles in large dendrites. Post-hoc analysis showed that CIS Oxy-females compared to CIS Sal-females had decreased density of cytoplasmic (p = 0.0006) and total (p = 0.0002) DOR-SIG particles in large CA1 dendrites (Fig. 9C).

A significant main effect of treatment was observed in the partitioning ratio of DOR-SIG particles localized to the plasma membrane of large CA1 dendrites (F_3,334_ = 4.1734; p = 0.0418). Post-hoc analysis showed that CIS Sal-females compared to CIS Sal-males had an increased ratio (F_1,154_ = 4.24; p = 0.0413) of DOR-SIG particles on the plasma membrane in large CA1 dendrites (Fig. 9E,F).

#### 3.3.4 CIS Sal- and Oxy-males had fewer DOR-labeled dendritic spines compared to CIS Sal- and Oxy-females

The percent of DOR labeled spines and distribution of DOR-SIG particles in dendritic spines from CIS rats in Cohort 3 are shown in Table 1. CIS Sal-females have a greater percentage of DOR-labeled spines in CA1 compared to CIS Sal-males (F_1,4_ = 112.5; p = 0.0004). Qualitatively, DOR-SIG particles are primarily found on the membrane and in the cytoplasm in CIS Sal-females, whereas DOR-SIG particles are found predominantly in the cytoplasm of CIS Sal-males. The percent of DOR-labeled spines remained higher in CIS Oxy-females compared to CIS Oxy-males. In the CIS Oxy-females, more DOR-SIG particles were in the cytoplasm than at the synapse or on the plasma membrane.

## 4 Discussion

**Fig. 10:**
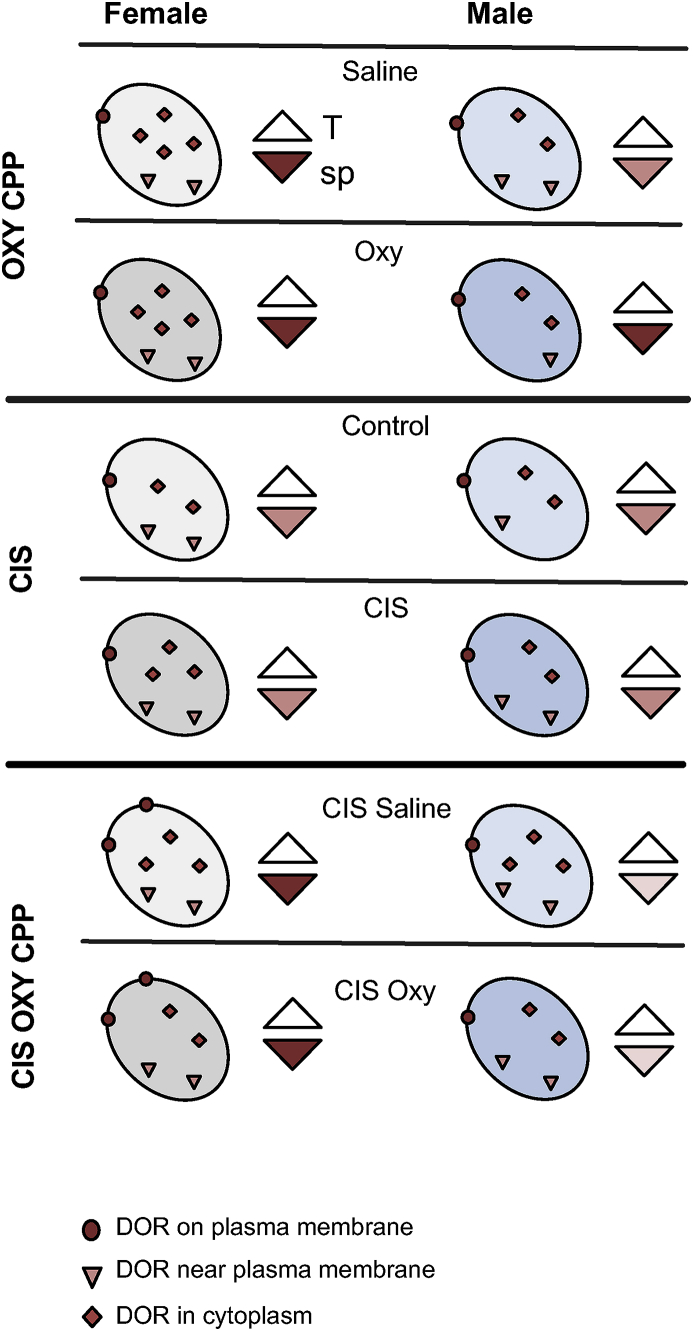
**Schematic diagram summarizing the distribution of DORs in CA1 pyramidal cell dendrites following Oxy CPP, CIS and CIS plus Oxy CPP**. Oval = dendritic shaft; Top white triangle = terminal (T); bottom colored triangle = dendritic spine (sp). The intensity of the red color in the triangles = the relative density of labeling. Oxy CPP (cohort 1): Sal-females compared to Sal-males have more cytoplasmic and total DORs in CA1 dendrites as well as more DOR-labeled spines, suggesting an enhanced sensitivity to DOR agonists. Following Oxy CPP, DORs redistribute from dendritic shafts to spines in males to densities similar to females. These changes in DOR distribution are consistent with those in CA3 dendrites (Ryan et al., 2018). CIS only (cohort 2): Control females compared to control males have greater near plasma membrane DORs in CA1 dendrites. Following CIS, cytoplasmic DORs in CA1 dendrites are elevated in females and near plasma membrane DORs are elevated in males. However, females still have more cytoplasmic DORs after CIS, suggesting greater reserve pools of DORs after CIS. CIS + Oxy CPP (cohort 3): CIS Sal-females compared to CIS Sal-males had more DORs on the plasma membrane of CA1 dendrites and in spines. Only CIS females acquired Oxy CPP. After Oxy, the distribution of DORs did not change in either females or males. This suggests that, similar to CA3 pyramidal cells (Reich et al., 2019), CIS primes the CA1 dendrites in females for sensitivity to DOR agonists. (For interpretation of the references to color in this figure legend, the reader is referred to the Web version of this article.)

Consistent with our prior findings in CA3 and DG from unstressed rats (Ryan et al., 2018), the present study shows that Oxy CPP results in sex-dependent changes in the subcellular distribution of DORs in CA1 pyramidal cells in a manner that would promote plasticity processes (Fig. 10). Moreover, the present study shows that CIS coupled with behavioral challenges redistributes DORs in CA1 in females, but not males, to prime for sensitivity to DOR agonists. Thus, together with prior studies in CA3 and DG (Bellamy et al., 2019; Reich et al., 2019), these studies suggest that the lack of changes in the opioid system seen in male rats following CIS may contribute to their diminished capacity to acquire Oxy CPP.

### 4.1 Methodological considerations

The tissue from the three cohorts of rats was processed for immunocytochemistry at different times, thus statistical comparisons in DOR-SIG particle density between the cohorts could not be made (Milner et al., 2011). Moreover, the handling and environment in the unstressed controls in cohorts 1 and 2 differed from one another; cohort 1 controls were injected with Sal and exposed to the CPP apparatus (Ryan et al., 2018), while cohort 2 controls were gently handled ~5–10 min per day for 10 days and returned to their home cage (Mazid et al., 2016; Milner et al., 2014). Prior studies have shown that Sal injections in male rats compared to unhandled controls are associated with increased spine density in hippocampus CA1 pyramidal cells (Horner et al., 1991). Further, exposure of the rats to the Oxy-CPP apparatus would have enriched their environment and thus may have affected total spine density and/or the number of subtypes of spines on CA1 dendrites (Kitanishi et al., 2009; Moser et al., 1997). Additionally, different anesthetics were used to euthanize the rats in cohort 1 (ketamine/xylazine) and cohort 2 (sodium pentobarbital) which could have affected the DOR trafficking (Beurel et al., 2016). These differences in handling, environment and/or anesthetic may have contributed to the baseline differences seen in DOR labeled spines and/or DOR densities in dendrites from control groups in cohorts 1 and 2. In contrast to control rats, few differences were seen in the subcellular distributions of DOR-SIG particles between the CIS rats (handled only) in cohort 2 and the CIS Sal-group (injected plus CPP apparatus) in cohort 3. This finding suggests that alterations in trafficking of DORs induced by CIS may override changes induced by handling, enriched environment and/or anesthetic. This would be consistent with the idea that a system adapts to chronic stress resulting in a new equilibrium set point (McEwen and Akil, 2020).

### 4.2 Unstressed females have greater dendritic DOR densities than males

Unstressed Sal-females (cohort 1) compared to unstressed Sal-males had greater DOR densities in the cytoplasm and in total in CA1 dendrites (Fig. 10). Similarly, unstressed control females (cohort 2) compared to unstressed control males had greater densities of near plasmalemmal DORs in CA1 dendrites. The current findings in estrus females are consistent with our previous study (Williams et al., 2011b) showing that proestrus females had higher DOR densities in small CA1 dendrites than males. Although the DORs were elevated in different dendritic compartments in the females from cohorts 1 and 2, these findings suggest that at baseline females have higher reserve pools of DORs. These non-synaptic reserve pools permit the rapid supply of transmembrane receptors that are important for potentiating synapse function (Kneussel and Hausrat, 2016).

The finding that unstressed control females compared to males (cohort 2) have higher near-plasmalemmal DORs in CA1 pyramidal cell dendrites is congruent with previous observations in CA3 pyramidal cell dendrites (Mazid et al., 2016). However, our previous studies with cohort 1 (Ryan et al., 2018) found that Sal-males compared to Sal-females had greater near-plasmalemma and total DOR densities in CA3 pyramidal cell dendrites. As the tissue samples from CA1 and CA3 were from the identical experiment, these differences cannot be attributed to methodological procedures. Thus, it is likely that these differences in DOR distribution are due to sex differences in the sensitivity of the CA1 vs. CA3 neurons to handling and/or environmental effects. This idea is supported by studies showing that sex can differentially affect synaptic plasticity responses and dendritic structure in both CA1 and CA3 pyramidal neurons (McLaughlin et al., 2005; Scharfman and MacLusky, 2017).

### 4.3 Following Oxy CPP, DORs redistribute to dendritic spines in males

Following Oxy CPP, the density of DORs in CA1 dendrites as well as the percentage of DOR-labeled spines remained unchanged in the females (Fig. 10). In contrast, DORs redistributed from the plasmalemmal associated compartments of CA1 dendrites to the dendritic spines in Oxy-males relative to Sal-males. Thus, while the DOR densities in CA1 pyramidal cell dendrites are still higher in Oxy-females compared to Oxy-males, the percentage of DOR-labeled spines in females and males after Oxy CPP is equivalent. These observations are similar to the DOR redistributions seen in CA3 pyramidal cell dendrites in both sexes following Oxy CPP (Ryan et al., 2018).

In addition to changes in DOR densities, our prior studies have shown that genes for neuroplasticity and related signaling molecules in CA1 are upregulated following Oxy CPP in a sex-dependent manner (Randesi et al., 2019). In particular, Oxy-females have elevated expression for *Akt* (AKT serine/threonine kinase 1), a signal transduction intermediate that is crucial for synaptic protein translation, including post-synaptic density-95 (Akama and McEwen, 2003; Brunet et al., 2001; Chong et al., 2005). Moreover, phosphorylated AKT is elevated in the cytoplasm of CA1 dendrites and spines in proestrus females compared to males (Znamensky et al., 2003). Oxy-males have elevated expression of activity regulated cytoskeletal-associated protein (*Arc)* (Randesi et al., 2019), an immediate early gene targeted to synapses [reviewed in (Bramham et al., 2010)] and brain-derived neurotrophic factor (*Bdnf*) which is involved in synaptogenesis (Cunha et al., 2010). *Arc* and *Bdnf* are especially important in LTP processes (Cunha et al., 2010; Plath et al., 2006). Oxy-males also have elevated mitogen activated protein kinase 1 *(Mapk1)*, a signaling molecule critical for integrating membrane receptor signals to the nucleus (Sananbenesi et al., 2003). *Mapk* signaling can affect several plasticity processes in hippocampus including glutamatergic receptor phosphorylation and LTP (Bi et al., 2000). The upregulation of these genes coupled with the presence of DORs on dendritic spines in both females and males would be expected to promote synaptic excitation and plasticity processes (Bao et al., 2007).

Like CA3 pyramidal cells and DG cells, CA1 pyramidal cells are innervated by an intrinsic opioid containing pathway (Gall et al., 1981). LEnk-containing entorhinal afferents via the TAT project to the region of the outer apical dendrites of CA1 pyramidal cells (Empson and Heinemann, 1995; Gall et al., 1981). In this region, studies in mice have shown that DORs regulate TAT activated feedforward inhibition in GABAergic interneurons (Rezai et al., 2013). In basal conditions DORs are predominantly on interneurons and, to a lesser extent on pyramidal neurons (Drake et al., 2007; Williams et al., 2011b). As neuropeptides like enkephalin are released non-synaptically (Thureson-Klein and Klein, 1990), the increased presence of DORs on dendritic spines seen in both Oxy-females and males have an increased probability of CA1 pyramidal cell activation. The presence of DORs on CA1 pyramidal cells may become even more important following opioid abuse. In particular, chronic abuse of morphine can lead to desensitization of MOR responses (Bailey and Connor, 2005) and thus DORs would become the primary target of exogenous opioids in CA1.

### 4.4 CIS paired with behavioral enrichment primes CA1 neurons for DOR-mediated plasticity

CIS alone (cohort 2) increased the reserve pools of DORs in CA1 pyramidal cell dendrites in both females (cytoplasm) and males (near plasmalemma) (Fig. 10). Although CIS alone did not change the percentage of DOR-labeled spines on CA1 pyramidal cells in either sex, when CIS was paired with Sal injections and placement in the CPP apparatus, the percentage of DOR-labeled spines increased in females. A similar increase in the percentage of DOR-labeled spines in females, but not males, was observed in CA3 dendrites following Sal injections and exposure to the CPP apparatus in our prior study on these same animals (Reich et al., 2019). Moreover, as in CA3 neurons (Reich et al., 2019), CIS paired with Oxy injections and CPP did not result in any further changes in the distributions of DORs in CA1 dendrites or spines in either females or males. Thus, Sal injections plus the enrichment of the exposure to the CPP behavior essentially “primed” the CA1 pyramidal cell dendrites for responses to exogenous opioids. Taken together with our previous studies (Bellamy et al., 2019; Reich et al., 2019; Ryan et al., 2018), these findings suggest that the reduced availability of DORs on CA1 and CA3 pyramidal cell dendritic spines as well as in plasmalemmal associated DORs on hilar GABAergic interneurons would decrease opioid mediated plasticity in multiple circuits within the hippocampus in males, but not in females.

Sex differences in the redistribution of DORs in CA1 dendrites following CIS are likely due to multiple factors. Our prior studies demonstrated that CA1 pyramidal cell dendrites colocalize DORs and corticotropin-releasing factor receptor type 1 (CRFR1) (Williams et al., 2011a) and that CRFR1 is about twice as dense in CA1 neurons from unstressed females compared to unstressed males (McAlinn et al., 2018). Following CIS, CRFR1 densities in CA1 dendrites are unchanged in females but increase in both the near plasmalemmal and cytoplasm of DOR dendrites in males (McAlinn et al., 2018). *In vitro* studies show that DOR activation attenuates CRF receptor mediated increases in intracellular cyclic AMP levels (Williams et al., 2011a). Thus, CIS likely alters the balance of DOR/CRFR1 signaling in favor of CRFR1 within the CA1 dendrites of males, but not in females. This assertion is supported by our findings (Randesi et al., 2018) that hippocampal tissues containing CA1 in CIS male rats, but not CIS female rats, have elevated CRF expression but reduced expression of genes important for opioid and neuroplasticity signaling [e.g., *Akt1*, cadherin-2 (*Cdh2*), neurotrophic receptor tyrosine kinase 2 (*Ntrk2*) and arrestin beta 1 (*Arrb1*)].

## 6 Conclusion

The present study demonstrates that following Oxy CPP, DORs within CA1 pyramidal cell dendrites remain positioned in unstressed female rats to enhance sensitivity to DOR agonists and traffic to dendritic spines in Oxy males where they could promote plasticity processes. We also find that following CIS and behavioral enrichment, DORs are redistributed to CA1 pyramidal cell dendritic spines of females to prime for enhanced sensitivity to DOR agonists. Conversely, CIS in males reduces DORs in CA1 pyramidal cell dendritic spines, which would then decrease sensitivity to DOR agonists. The CA1 region is important for spatial memory and chronic stress is known to impair spatial reference memory in males, but not females [reviewed in (Conrad, 2010; McEwen and Milner, 2017)]. Thus, the inability of DORs to increase in dendritic spines in males following CIS may contribute to their diminished capacity to acquire Oxy CPP.

These sex differences in DOR distributions in CA1 following Oxy CPP in both unstressed and CIS rats are similar to what we observed in our prior studies in CA3 and the DG (Mazid et al., 2016; Reich et al., 2019; Ryan et al., 2018). Together, they demonstrate that DORs as well as MORs are redistributed in at least three hippocampal circuits in a manner that would facilitate plasticity processes important for opioid-associated learning, especially in females. Moreover, after CIS, these processes are still in place in all three hippocampal opioid circuits in females. This protection of the opioid system in female rats following CIS may contribute to their greater susceptibility to opioid addiction and reinstatement (Becker et al., 2017).

## Author contributions

T.A.M., M.J.K. and B.S.M. funding acquisition and designed research; B.R.R., M.J., J.M.B., E.G., V.P., N.H.C., A.G.D., Y.Z., T.A.M., J.D.G. and E.M.W. performed research; B.R.R., M.J., J.M.B., N.H.C. analyzed data; B.R.R., E.M.W., T.A.M., B.S.M.*, M.J.K. wrote the paper. *abstract and first draft.

## CRediT authorship contribution statement

**Batsheva R. Rubin:** Formal analysis, Data curation, Writing - original draft, Writing - review & editing. **Megan A. Johnson:** Formal analysis, Data curation. **Jared M. Berman:** Formal analysis, Data curation. **Ellen Goldstein:** Data curation, Formal analysis. **Vera Pertsovskaya:** Data curation, Formal analysis. **Yan Zhou:** Funding acquisition. **Natalina H. Contoreggi:** Formal analysis, Data curation. **Andreina G. Dyer:** Data curation. **Jason D. Gray:** Funding acquisition, Writing - original draft. **Elizabeth M. Waters:** Funding acquisition, Writing - original draft. **Bruce S. McEwen:** Funding acquisition, Conceptualization, Writing - original draft. **Mary Jeanne Kreek:** Funding acquisition, Conceptualization, Writing - original draft, Writing - review & editing. **Teresa A. Milner:** Funding acquisition, Conceptualization, Formal analysis, Data curation, Writing - original draft, Writing - review & editing.

## Declaration of competing interest

The authors declare no competing financial interests.
